# Impact of High Hydrostatic Pressure and Soaking Solutions on the Surface Structure, Carotenoids, Individuals' Phenolic Acids and Flavonoids Profile of Orange‐Fleshed Sweet Potato Flour

**DOI:** 10.1002/fsn3.70300

**Published:** 2025-05-18

**Authors:** Muhammad Azeem, Ramsha Nasir, Safeena Amjad, Naveed Hussain, Kiran Islam, Syed Abdul Wadood, Isam A. Mohamed Ahmed, Noman Walayat, Ildephonse Habinshuti

**Affiliations:** ^1^ Faculty of Food Technology and Nutrition Sciences Lahore University of Biological and Applied Sciences Lahore Pakistan; ^2^ Department of Nutrition and Health Promotion University of Home Economics Lahore Pakistan; ^3^ Department of Applied Sciences (FEST) Hamdard University Karachi Pakistan; ^4^ School of Food Science and Pharmaceutical Engineering Nanjing Normal University Nanjing China; ^5^ Department of Food Science and Nutrition, College of Food and Agricultural Sciences King Saud University Riyadh Saudi Arabia; ^6^ College of Tea Science and Tea Culture Zhejiang Agriculture and Forestry University Hangzhou China; ^7^ School of Agriculture and Food Sciences, College of Agriculture, Animal Sciences and Veterinary Medicine University of Rwanda Musanze Rwanda

**Keywords:** carotenoids, flavonoid compounds, high hydrostatic pressure, phenolic compounds, soaking solution, sweet potato orange fleshed

## Abstract

The main objective of the current work was to monitor the impact of soaking solution (citric acid, calcium chloride and ascorbic acid) and high hydrostatic pressure (HHP) (100, 200 and 400 MPa) on the carotenoids, antioxidant activity (ORAC, Oxygen Radical Absorbance Capacity), surface structure, and individuals' phenolic acids and flavonoids of orange‐fleshed sweet potato flour (OFSP). Carotenoids in OFSP increased several folds during treatment with ascorbic acid and HHP application at 400 MPa, whereas decreased contents were observed with the treatment of calcium chloride and citric acid. Ascorbic acid and 400 MPa significantly increased the phenolic acids (28.02, 27.40%) and flavonoids (8.45, 63.57%) respectively. The most abundant phenolic acid and flavonoid in OFSP were 3,5‐di‐O‐caffeoylquinic acid and Quercitrin, respectively. Pearson correlation coefficient analysis was performed between ORAC and phenolic acids such as 5‐Ocaffeoyl quinic acid (0.90), 4‐O‐caffeoyl quinic acid (0.89) and Myricetrin (0.86) which proved explicitly that antioxidant activity was mainly associated with these compounds. It was concluded that ascorbic acid and 400 MPa treatments of OFSP significantly impacted the increased levels of phenolic acids, flavonoids, and carotenoids. OFSP treated with ascorbic acid and 400 MPa might be used for multiple products with desired nutrients.

## Graphical abstract

High hydrostatic pressure (HHP) and soaking solution modified the properties of orange‐fleshed sweet potato flour (OSPF). Carotenoids in OSPF increased several folds during treatment with ascorbic acid and HHP application at 400 MPa. Ascorbic acid and 400 MPa significantly increased phenolic acids (28.02, 27.40%) and flavonoids (8.45, 63.57%) respectively. The most abundant phenolic acid in OSPF was 3,5‐di‐O‐caffeoylquinic acid, whereas among flavonoids was Quercitrin.

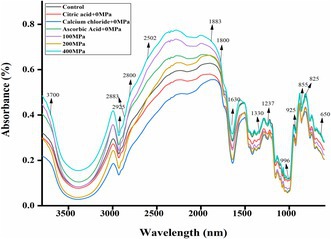

## 1 Introduction

Many emerging nations are facing problems of extensive population and shortage of cultivated land. These emerging needs encouraged scientists to investigate underused plants for human consumption, exclusively in places where staple crops such as wheat, maize, and rice are hard to cultivate owing to the scarcity of water (Albahri et al. 2023). Moreover, sweet potatoes possess a variety of attributes such as capability to adapt to tough seasonal conditions and short production cycle (Azeem et al. 2019). Among different cultivars of sweet potatoes, orange‐fleshed sweet potato flour (OFSP) was found to be a rich source of β‐carotene and phenolic compounds (Arebo et al. 2023). OFSP exhibited excellent antioxidant activity owing to the presence of its conjugated double bonds (Azeem et al. 2024).

Sweet potatoes exhibited abundant quantities of phenolic compounds as compared to commercial vegetables (Kaur and Kapoor 2001). Phenolics belong to secondary metabolites with low molecular weight compounds that exist in land plants. Phenolic compounds substantially influence the taste of food such as the pungency of chilies and the astringent taste of beer. Phenolic compounds are also building blocks of dietary fiber and lignin, which are mainly responsible for the texture and nutritional value of vegetables. Phenolic compounds impart color to the food and possess multiple biological benefits such as strong antihypertensive, anti‐inflammatory, anticarcinogenic, and deterrence of cholesterol‐induced atherosclerosis (Kim et al. 2015; Cai et al. 2016; Kita et al. 2013; Truong et al. 2007). Delay in the degradation of phenolic compounds is necessary for the benefits to both consumers and processors (Martynenko and Chen 2016). OFSP was selected for the study of phenolic compounds due to its good absorption and maintenance of the original form during processing (Kim et al. 2012).

OFSP is reported to be used in the preparation of value‐added products, such as color beverages, dairy foods, bread, noodles, and flakes (Gras et al. 2017; Neela and Fanta 2019). The main issues in the above‐mentioned products are enzymatic and non‐enzymatic reactions during processing. Enzymatic reactions may take place even at low temperatures due to peroxidase and polyphenol oxidase, whilst non‐enzymatic reactions (Maillard reaction) occur during hot conditions as a result of reactions between reducing sugars and amino acids (Graham‐Acquaah et al. 2014). However, various soaking solutions, including sodium metabisulphite and citric acid, have already been used to overcome the aforementioned issues (Kuyu et al. 2018; Ahmed et al. 2010). Furthermore, when food is subjected to thermal processing, the composition of bioactive compounds and other quality attributes of products significantly changes (Kim et al. 2012).

High hydrostatic pressure (HHP) (non‐thermal processing technology) has gained a lot of recognition in the food industry owing to the inactivation of endogenous enzymes and prevention of color loss (Peng et al. 2016; Liu et al. 2013). HHP technology has proactively been used for different food items such as vegetables, fruits, meat, seafood, and fish, etc. (Nabi et al. 2021). Various studies have already verified that HHP significantly influenced the phenolic compounds in tomato puree (Jeż et al. 2018), aronia juice (Błaszczak et al. 2017), *lonicera japonica* Flos (Duan et al. 2018), apricot nectars (Huang et al. 2013) and red wine (Sun et al. 2016). However, no study has been published regarding the influence of HHP and soaking solution on the structure, phenolic acids, flavonoids, carotenoids, and antioxidant activity of OFSP. In the current study, Pushu 32 was selected as it belongs to the most prominent variety in China (Azeem et al. 2019; Kourouma et al. 2019b, 2019a). Therefore, it is necessary to explore proper processing techniques having negligible impact on the degradation of OFSP quality. The availability and stability of phenolic compounds after HHP and soaking solution of OFSP are also necessary to get a significant impact on human health.

Hence, the current study attempted to identify the structure as well as elucidate the influence of HHP and soaking solutions on the retention properties of carotenoids, antioxidant activity, individual phenolic acids, and flavonoids in OFSP.

## 2 Materials and Methods

### 2.1 Materials

Orange‐fleshed sweet potato (Pushu 32) was bought from Hebei Agriculture Research Center (China). Ethanol, phosphoric acid, acetonitrile, trifluoroacetic acid, ethyl acetate, formic acid, methanol, and L‐ascorbic acid were bought from Sigma‐Aldrich Inc. (St. Louis, MO, USA). Remaining other reagents and chemicals were of analytical grade.

### 2.2 High Hydrostatic Pressure (HHP) and Soaking Solution

Sweet potatoes were washed to dislodge the adhered particles and were converted into slices of 4 mm in thickness. These slices (250 g) were hermetically vacuum sealed in Nylon/LLDPE polymeric pouches (diameter 16 × 25 cm length). High hydrostatic pressure was applied using HHP machine (HHP.L3‐600/0.6; Huatai Senmiao Engr. & Tech. Ltd. Co., Tianjin, China). Water was used inside the machine as a thermal regulator and for the transfer of pressure. Vacuum‐packed samples were subjected to isostatic pressure of (100, 200, 400 MPa, respectively) for 6 min with a compression gradient of 5 MPa/s at ambient temperature. Sweet potato slices were also separately treated with soaking solutions. The slices were submerged in soaking solutions (1% (w/v) each of ascorbic acid, calcium chloride, and citric acid) for 15 min under ambient conditions. Successively, the immersed samples were drained, followed by drying at 60°C. Dried samples were ground and sieved through a 0.45 mesh size sieve for further use.

### 2.3 Fourier Transform Infrared Spectroscopy (FTIR)

FTIR of OFSP was performed using the Nicolet 6700‐Fourier transform infrared spectrometer (Canada). The following parameters were used as mentioned below:

Sample preparation: The OFSP sample was mixed with KBr (1:60 w/w) in the Lab ball mill to ensure homogeneity.

Pellet formation: Above mixture was pressed into a pellet by using a laboratory press (pressure of 10 tones) in.

Measurement: Ready pellet adjusted in holder dedicated accessory of the spectroscopy was used for measurement.

Spectral range: Exclusive range of 500–4000 cm^−1^ was utilized with a resolution of 2 cm^−1^.

Detector: A deuterated triglycine sulfate detector was used for calculating the spectra.

### 2.4 Phenolic Acids Quantification Through RP‐HPLC

Qualitative and quantitative analysis of phenolic acids in the extract of OFSP were carried out through reverse‐phase high‐performance liquid chromatography (RP‐HPLC) (Agilent Technologies, USA) by following the method of Makori et al. (2020). Concisely, 1 g of sample was blended in 20 mL of 70% ethanol and treated ultrasonically for 35 min at 45°C. Subsequently, the blend was centrifuged at 5500 *g* for 15 min to collect the supernatant, which was concentrated through a vacuum evaporator. Quantification and identification were carried out by comparison with standards. The results of phenolic acids were presented as mg/g DW.

### 2.5 Quantification of Flavonoids Compounds Through HPLC

The liquid–liquid extraction was used to separate the polarities through different organic solvents. Crude extract of OFSP was concentrated through vacuum until half of the volume was left. Then extraction was done with equal volume of petroleum ether for the removal of chlorophyll as well as impurities (soluble in lipids). Subsequently, the resulting extract was mixed with ethyl acetate in order to separate the water phase. Finally, the ethyl acetate phase was concentrated through vacuum until dryness. Thereafter, flavonoid compounds present in the extracts of OFSP were quantified through High‐performance liquid chromatography HPLC (Shimadzu, Japan), consisting of a UV detector (SPD‐20A), liquid infusion unit (LC‐20AB), automatic sampler (SIL‐20 AC), degasser (DGU‐20A3), C18 column (4.6 × 150 mm, 5 μm, inertsil ODS‐SP, Shimadzu), column oven (CTO‐20 AC) and control unit (CBM‐20A). The mobile phase consisted of (A) 0.5 (v/v) phosphoric acid and (B) 100% acetonitrile. The flow rate was adjusted to 1.0 mL/min with an injection volume of 20 μL, and the detection wavelength was set at 326 nm. The elution procedure was managed as 0–15 min, 20%–65% B; 15.0–15.1 min, 65%–80% B; 15.1–20.0 min, 80% B; 20.0–20.1 min, 80%–20% B; and 20.1–25.0 min, 20% B. Quantification and identification were carried out by comparison with standards. The amount of each flavonoid compound was presented as mg/g DW.

### 2.6 Carotenoids

Assay concerned with carotenoids was performed in accordance with the protocol as explained by de Carvalho et al. (2012). Each sample (1 g) was extracted with petroleum ether and acetone 30 mL (80:20) using ultrasonic treatment for 10 min. The extraction procedure was repeated until residues became colorless to ensure complete extraction. Then, centrifugation was carried out at 5000 g for 15 min. The water phase was discarded with a pinch of 3 g anhydrous sodium sulfate. Supernatants were concentrated through a rotary evaporator at 85 rpm for 30°C, followed by the addition of 25 mL of petroleum ether (100%) to collect the entire sample. The absorbance of the solution was determined at 450 nm using a UV1101 spectrophotometer (Hitachi, Japan). The results of total carotenoids were expressed as μg/g DW. The total carotenoid content was calculated using the following formula:

$$\text{Crotenoid contents}\;\left(\mathrm{\mu g}/g\right)=\frac{A\times V\left(\mathrm{mL}\right)\times 10^{4}}{\begin{array}{c} A^{1\%}\\ 1\mathrm{cm} \end{array}\times P\left(g\right).}$$

Where *A* = Absorbance; *V* = Total extract volume; *p* = sample weight $\begin{array}{c} A^{1\%}\\ 1\mathrm{cm} \end{array}$ =2592 (β‐carotene Extinction Coefficient in petroleum ether).

### 2.7 Oxygen Radical Absorbance Capacity (ORAC)

ORAC assay was executed in accordance with the method of Thaipong et al. (2006). Briefly, sample extract (20 μL), phosphate buffer (20 μL, 75 mM, pH 7.4), AAPH (140 μL consisting of 18.28 mmol/L), and sodium fluorescein solution (63 mmol/L) were evenly mixed using a vortex mixer. Subsequently, the mixer was inserted into a 96‐well microplate followed by incubation at 37°C for 15 min. Successively, fluorescein was investigated at 485 (excitation) and 535 nm (emission) using a multifunctional microplate reader (Chameleon, Hidex, Turku, Finland) at 37°C for 30 min. The findings were presented as μmol of Trolox equivalent (TE)/g DW.

### 2.8 Statistical Analysis

Statistical analysis was carried out using SAS version 8.1 software (SAS Institute Inc., Cary, NC, USA). The results were depicted as mean ± standard deviation. *p* < 0.05 was considered a statistical significance. Pearson correlation coefficient was used to determine the relationship between antioxidants and phenolic compounds. All measurements and analysis were performed in triplicate.

## 3 Results and Discussion

### 3.1 FTIR

**FIGURE 1:**
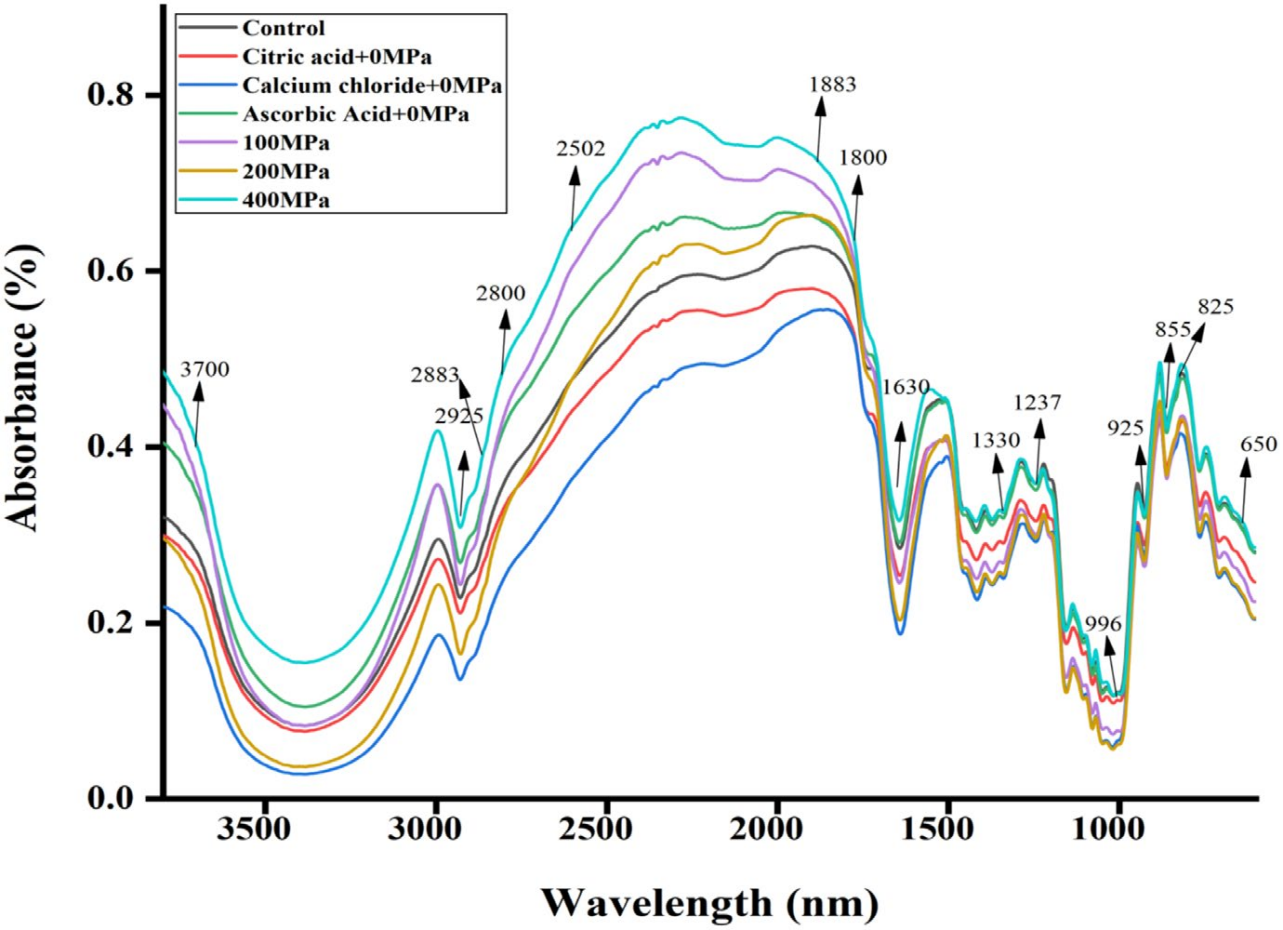
FTIR spectra of orange‐fleshed sweet potato flour influenced by thesoaking solution and high hydrostatic pressure.

Figure 1 shows FTIR spectra of OFSP as influenced by soaking solution and HHP. FTIR spectroscopy was possibly utilized as a quality control in food, and spectral lines exhibited the chemical groups of the components present in the samples. FTIR spectra of OFSP exhibited the typical bands in two regions, that is, 650–1800 cm^−1^ and 2800–3700 cm,^−1^ which were associated with protein and carbohydrates, respectively. The bands in the region of 855 and 925 cm^−1^ are associated with C—C skeletal vibration and glycosidic bonds of the starch, respectively (Olga et al. 2019). The band at 996 cm^−1^ has been allied with the crystalline structure of starch and hydroxyl groups in intermolecular hydrogen bonding (Warren et al. 2016). Furthermore, an absorption band at 1237 cm^−1^ was associated with the CH_2_OH related mode and C—O—H deformation (Kizil et al. 2002). FTIR spectra accurately determine the ash content within the peak values of 835–2502 nm with 8 cm^−1^ wave number revolution and 200 scans/spectrum (Amir et al. 2013). Few authors reported that the 1630 cm^−1^ peak was concerned with the water absorption in the amorphous region of starch. The band observed at 2925 and 2883 cm^−1^ indicated the presence of CH_2_ groups and probably related to the C—H stretching. However, changes in intensity are possibly attributed to variations in amylose and amylopectin present in OFSP (Cui and Zhu 2019).

Soaking solution treatment and HHP application of OFSP exhibited a pronounced impact on FTIR spectra. OFSP soaked in calcium chloride and citric acid solutions exhibited a decreasing trend of absorbance in all bands. A few bands shifted to others, and some new peaks with structural modification have been observed. Such types of structural changes might be due to the attachment of calcium chloride and citric acid with the polymeric chains of carbohydrates and proteins, thereby replacing polymer‐polymer interactions that prevent the forces (Van der Waals and hydrogen bonding) responsible for the integrity of polymer molecules together. Application of HHP influenced the intensity of peaks (increase the peak height) owing to the alteration of molecular bonding of the components. It may also be assumed that the application of HHP possibly influenced the intensity of the bands due to the association of crystallization and retrogradation of starch. The higher absorbance of FTIR spectra might also be associated with an increase in water absorption capacity (Warren et al. 2016).

### 3.2 Quantification of Phenolic Acids Through RP‐HPLC

Phenolic acids present in the extracts of OFSP treated with HHP and soaking solutions are presented in Table 1. Eight phenolic compounds were identified through comparison with different retention times after running their standards under similar conditions, and further quantifications of compounds were done from their respective regression equations as presented in Table 2. The order of quantification of eight phenolic acids observed in the extract of OFSP was as follows: 3,5‐di‐O‐caffeoylquinic acid > 3‐O‐caffeoylquinic acid > 5‐O‐caffeoylquinic acid > 3,4‐di‐O‐caffeoylquinic acid > 4‐O‐caffeoylquinic acid > 3,4,5‐tri‐O‐caffeoylquinic acid > caffeic acid > 4,5‐di‐O‐caffeoylquinic acid.

**TABLE 1:** Contents of phenolic acids in orange‐fleshed sweet potato flour as influenced by high hydrostatic pressure and soaking solution (mg/g DW).

Treatments	5‐Ocaffeoylquinic acid	3‐O‐caffeoylquinic acid	4‐O‐caffeoylquinic acid	Caffeic acid	4,5‐di‐O‐caffeoylquinic acid	3,5‐di‐O‐caffeoylquinic acid	3,4‐di‐O‐caffeoylquinic acid	3,4,5‐tri‐O‐caffeoylquinic acid	Total
Control	0.83 ± 0.11d	0.72 ± 0.07e	0.50 ± 0.03c	0.026 ± 0.001d	0.022 ± 0.009b	1.60 ± 0.05a	0.76 ± 0.01d	0.08 ± 0.002b	4.55 ± 0.32c
Citric acid + 0.1 MPa	0.71 ± 0.01e	0.46 ± 0.02f	0.47 ± 0.01d	0.025 ± 0.001d	0.019 ± 0.003bc	0.88 ± 0.02e	0.62 ± 0.03f	0.05 ± 0.001d	3.17 ± 0.61d
Calcium chloride + 0.1 MPa	0.76 ± 0.01de	0.86 ± 0.03d	0.30 ± 0.02f	0.032 ± 0.002c	0.031 ± 0.002a	0.73 ± 0.02f	0.89 ± 0.02c	0.05 ± 0.002d	3.65 ± 0.12d
Ascorbic acid + 0.1 MPa	1.96 ± 0.04a	1.37 ± 0.02b	0.75 ± 0.03a	0.036 ± 0.002b	0.011 ± 0.002c	0.89 ± 0.01d	0.74 ± 0.02d	0.06 ± 0.003c	5.83 ± 0.34a
100 MPa	1.25 ± 0.05b	1.24 ± 0.01c	0.56 ± 0.04b	0.035 ± 0.003b	0.014 ± 0.002bc	1.29 ± 0.02b	0.94 ± 0.01b	0.08 ± 0.001b	5.41 ± 0.61ab
200 MPa	0.99 ± 0.02c	1.37 ± 0.08b	0.38 ± 0.01e	0.030 ± 0.004c	0.016 ± 0.003bc	1.05 ± 0.03c	0.10 ± 0.01a	0.02 ± 0.002f	4.79 ± 0.73c
400 MPa	1.03 ± 0.03c	1.52 ± 0.09a	0.49 ± 0.01c	0.038 ± 0.005a	0.017 ± 0.001bc	1.60 ± 0.01a	0.99 ± 0.03a	0.10 ± 0.001a	5.84 ± 0.57b

*Note:* Values are the mean ± standard deviation (SD). Different letters within the same column indicate significantly different at *p* < 0.05.

**TABLE 2:** Retention time, absorbance, identified compounds and standard curve of phenolic acids and flavonoids found in orange‐fleshed sweet potato flour.

Compounds	Peak	Retention time (min)	HPLC λ_max_ (nm)	Identified compound	Linear calibration curve	*R* ^2^
Phenolic acids	1	1.72	326	5‐O‐caffeoylquinic acid	*y* = 12207x + 1324.2	0.9914
	2	2.10	326	3‐O‐caffeoylquinic acid	*y* = 6198.6x + 19535	0.9976
	3	3.18	326	4‐O‐caffeoylquinic acid	*y* = 26481x + 14793	0.9942
	4	5.46	326	Caffeic acid	*y* = 34469x − 5759.1	0.9951
	5	6.46	326	4,5‐di‐O‐caffeoylquinic acid	*y* = 35830x − 10319	0.9981
	6	8.54	326	3,5‐di‐O‐caffeoylquinic acid	*y* = 26069x − 52727	0.9968
	7	9.23	326	3,4‐di‐O‐caffeoylquinic acid	*y* = 966.15x + 465.53	0.9949
	8	10.26	326	3,4,5‐tri‐O‐caffeoylquinic acid	*y* = 37912x − 23973	0.9937
Flavonoids	1	5.91	326	Myricetrin	*y* = 30104x − 122802	0.9897
	2	7.32	326	Isoquercitrin	*y* = 37242x − 991142	0.9914
	3	7.95	326	Astragalin	*y* = 39421x − 218279	0.9913
	4	8.59	326	Quercitrin	*y* = 36234x − 129578	0.9909
	5	10.19	326	Tiliroside	*y* = 57391x − 245164	0.9901
	6	11.18	326	Quercetin	*y* = 31917x − 88198	0.9901

Significant quantities of 3,5‐di‐O‐caffeoylquinic acid (1.60 mg/g), 5‐O‐caffeoylquinic acid (0.83 mg/g), 3,4‐di‐O‐caffeoylquinic acid (0.76 mg/g) 3‐O‐caffeoylquinic acid (0.72 mg/g) and 4‐O‐caffeoylquinic acid (0.50 mg/g) were present in the extract of control samples (OFSP). Some other compounds such as Caffeic acid, 4,5‐di‐O‐caffeoylquinic acid, and 3,4,5‐tri‐O‐caffeoylquinic acid were present in very small quantities in the control samples of OFSP. The quantities of phenolic compounds in the extracts of OFSP increased through the treatment of ascorbic acid and application of HHP at 400 MPa. The increased quantities of phenolic compounds in the samples might be due to the inactivation of enzymes such as PPO (EC 1.10.3.1) and peroxidase (EC 1.11.1.x) (responsible for the oxidation of phenolic compounds) via application of ascorbic acid and HHP (Błaszczak et al. 2017). Moreover, polyphenols may also be increased during HHP application due to rupturing of cells in the extracts of OFSP (Tokuşoǧlu et al. 2010). The highest (5.84 mg/g) total quantities of phenolic compounds were recorded in the extracts of OFSP treated with 400 MPa, which accounted for 22.08% higher contents than control samples (OFSP).

In order to find the relationship between individual phenolic acids and antioxidant activity of OFSP, the Pearson correlation coefficient was performed. 5‐O‐caffeoylquinic acid presented the highest antioxidant activity, followed by 4‐O‐caffeoylquinic acid. The correlation between phenolic acids and antioxidant activity of OFSP recommended that the antioxidant activity of OFSP was mainly concerned with the 5‐O‐caffeoylquinic acid and 4‐O‐caffeoylquinic acid.

### 3.3 Quantification of Flavonoid Compounds Through RP‐HPLC

Flavonoid compounds present in the extracts of OFSP treated with HHP and soaking solutions are presented in Table 3. Six flavonoid compounds, that is, Myricetrin, Isoquercitrin, Astragalin, Quercitrin, Tiliroside, and Quercetin, were identified through comparison with different retention times after running their standards under similar conditions, and further quantifications of compounds were done from their respective regression equations as presented in Table 3. The order of quantification of the six flavonoid compounds observed in the extract of OFSP was as follows: Quercitrin > Astragalin > Isoquercitrin > Tiliroside > Quercetin > Myricetrin. Quercitrin was present in the highest concentration (0.75 mg/g) in the control samples (OFSP) whereas Quercitrin, Quercetin, Tiliroside, Isoquercitrin, and Myricetrin were present in lower concentrations. Flavonoid values of the OFSP sample increased with soaking solutions of ascorbic acid (1.08 mg/g DW), whilst decreased corresponded to calcium chloride with 0.1 MPa (0.96 mg/g DW). The values of flavonoids were recorded maximum at 400 MPa (1.72 mg/g DW), whereas minimum values were observed at 100 MPa (1.40 mg/g DW). HHP application enhanced the quantification that might be attributed to the breakdown of cell wall structure and matrix modifications, which possibly increase the liberation of these compounds (Tokuşoǧlu et al. 2010).

**TABLE 3:** Contents of flavonoids in orange‐fleshed sweet potato flour as influenced by high hydrostatic pressure and soaking solution (mg/g DW).

Treatments	Myricetrin	Isoquercitrin	Astragalin	Quercitrin	Tiliroside	Quercetin	Total
Control	0.033 ± 0.004a	0.065 ± 0.001b	0.121 ± 0.02ef	0.75 ± 0.02c	0.056 ± 0.001d	0.045 ± 0.002 g	1.07 ± 0.26b
Citric acid + 0.1 MPa	0.027 ± 0.006a	0.044 ± 0.003f	0.135 ± 0.01e	0.68 ± 0.01e	0.048 ± 0.002f	0.059 ± 0.002c	1.00 ± 0.23b
Calcium chloride + 0.1 MPa	0.037 ± 0.001a	0.055 ± 0.001d	0.183 ± 0.01c	0.58 ± 0.03f	0.046 ± 0.001f	0.05 ± 0.002f	0.96 ± 0.20b
Ascorbic acid + 0.1 MPa	0.071 ± 0.061a	0.041 ± 0.002f	0.163 ± 0.01d	0.69 ± 0.04d	0.054 ± 0.001e	0.052 ± 0.001e	1.08 ± 0.23b
100 MPa	0.034 ± 0.001a	0.052 ± 0.002e	0.203 ± 0.01b	0.97 ± 0.02b	0.069 ± 0.005b	0.068 ± 0.001b	1.40 ± 0.33ab
200 MPa	0.036 ± 0.003a	0.061 ± 0.001c	0.106 ± 0.01f	1.19 ± 0.03a	0.080 ± 0.002a	0.054 ± 0.001d	1.53 ± 0.42a
400 MPa	0.039 ± 0.001a	0.071 ± 0.001a	0.34 ± 0.02a	1.10 ± 0.01a	0.066 ± 0.001c	0.099 ± 0.001a	1.72 ± 0.38a

*Note:* Values are the mean ± standard deviation (SD). Different letters within the same column indicate significantly different at *p* < 0.05.

The sum of flavonoid compounds in the extract of control OFSP was 1.07 mg/g, whereas the highest quantities were noted in 400 MPa treated OFSP samples (1.72 mg/g) which were 65% higher than control OFSP samples. Quercitrin is the most abundant flavonoid compound in the samples of OFSP. In order to find the association between flavonoid compounds and the antioxidant activity of OFSP, the Pearson correlation coefficient was performed. Myricetrin showed the highest (0.86) correlation coefficient with the antioxidant activity. The correlation between the flavonoid compound and the antioxidant activity of OFSP recommended that the antioxidant activity of OFSP was mainly concerned with Myricetrin.

### 3.4 Carotenoids

**FIGURE 2:**
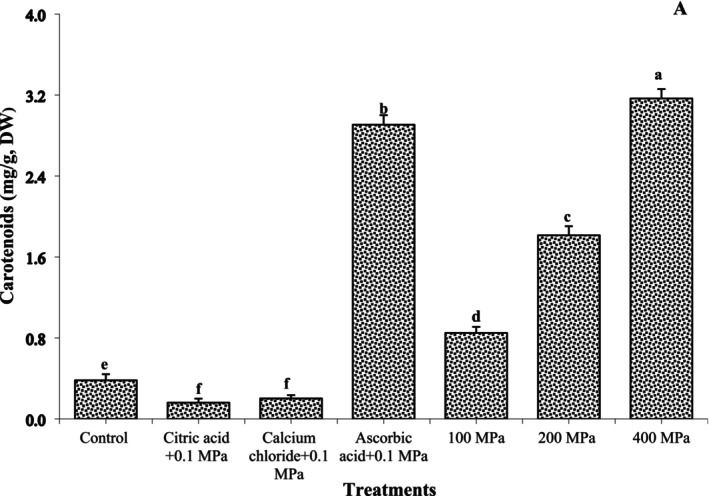
Carotenoids of orange‐fleshed sweet potato flour as influenced by the soaking solution and high hydrostatic pressure.

Carotenoids of OFSP as influenced by soaking solution and HHP are presented in Figure 2. Carotenoids of control OFSP sample were 0.38 mg/g DW. Carotenoids of OFSP sample increased with a soaking solution of ascorbic acid, whereas the decrease corresponded to citric acid and calcium chloride with 0.1 MPa. Carotenoids increased with the application of HHP; however, the maximum carotenoids were observed at 400 MPa (3.17 mg/g DW). Current findings suggested that total carotenoids exhibited better retention after HHP application. Hendrickx et al. (1998) evaluated that HHP application above 350 MPa enhanced the extraction process of carotenoids owing to the breakdown of the protein carotenoids complex. Furthermore, HHP application increased the extraction through the disruption of chromoplasts where carotenoids are located (Zuluaga et al. 2016).

### 3.5 ORAC

**FIGURE 3:**
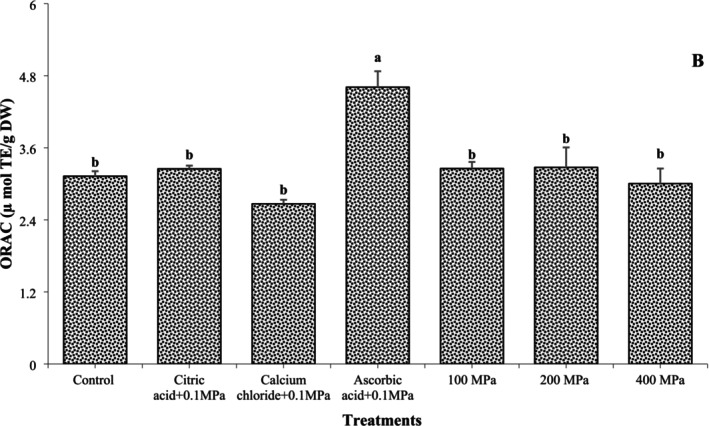
ORAC of orange‐fleshed sweet potato flour influenced by the soaking solution and high hydrostatic pressure.

ORAC of OFSP as influenced by soaking solution and HHP is exhibited in Figure 3. ORAC of control OFSP sample was 3.12 μmol TE/g DW. ORAC values of OFSP sample increased with soaking solutions of ascorbic acid, whereas a decrease corresponded to calcium chloride with 0.1 MPa. The values of ORAC were recorded higher at 100 and 200 MPa, whereas lower values were observed at 400 MPa as compared to control. Barba et al. (2012) also reported similar observations, that is, ORAC values of orange‐juice milk beverage were drastically increased at 100 MPa, whereas decreased at 400 MPa. The soaking solution of ascorbic acid in the current study showed a positive effect on ORAC values. Burguieres et al. (2007) investigated that ascorbic acid maintained bioactive compounds in the active state. Typically, HHP applications were thought to significantly influence the extraction of bioactive compounds. However, the influence of HHP on antioxidant activity varies in accordance with the time and temperature and food matrix (Keenan et al. 2011).

## 4 Conclusion

The present study provided sufficient, reproducible, and sensitive detection of phenolic compounds, carotenoids, and antioxidant activity. The analyzed parameters proved explicitly that pressure and soaking solution showed a significant influence on the investigated compounds. HHP application enhanced the intensity of the FTIR band that explicated increased protein and carbohydrate, which are possibly associated with crystallization and retrogradation of starch. The most abundant phenolic acid and flavonoids in OFSP were 3,5‐di‐O‐caffeoylquinic acid and Quercitrin, respectively. Application of ascorbic acid and 400 MPa enhanced the phenolic acids, flavonoids, and carotenoids. In the future for the pursuit of industrial application, expedited research should focus on elucidating the mechanism involved in the higher phenolic compound synthesis with ascorbic acid and 400 MPa that would possibly be useful for further increase of the nutraceutical value of sweet potatoes.

## Author Contributions

**Muhammad Azeem:** formal analysis (equal), methodology (equal), visualization (equal), writing – original draft (equal). **Ramsha Nasir:** formal analysis (equal), methodology (equal), visualization (equal), writing – original draft (equal). **Safeena Amjad:** data curation (equal), formal analysis (equal), validation (equal). **Naveed Hussain:** validation (equal), visualization (equal). **Kiran Islam:** investigation (equal), methodology (equal), validation (equal), visualization (equal). **Syed Abdul Wadood:** project administration (equal), validation (equal), visualization (equal). **Isam A. Mohamed Ahmed:** validation (equal), visualization (equal). **Noman Walayat:** validation (equal), visualization (equal). **Ildephonse Habinshuti:** validation (equal).

## Conflicts of Interest

The authors declare no conflicts of interest.

## Data Availability

We declare that the entire data supporting the findings of this study is available within the paper. Furthermore, according to the demand of the reviewer raw data will be provided in any format by (muhammad.azeem@ubas.edu.pk).
